# Preparation of a Lyophilized Bovine Milk Reference Material for Quality Control of Amoxicillin Detection

**DOI:** 10.3390/vetsci13020193

**Published:** 2026-02-15

**Authors:** Shulin Wei, Wei Zhang, Decheng Suo, Peilong Wang, Kang Ma, Ruiguo Wang

**Affiliations:** 1 Institute of Quality Standards and Testing Technology for Agro-Products, Chinese Academy of Agricultural Sciences, Beijing 100081, China; weishulin@caas.cn (S.W.); zhangwei@caas.cn (W.Z.);; 2 National Institute of Metrology of China, Beijing 100029, China

**Keywords:** amoxicillin, reference material, lyophilized bovine milk, isotope dilution–liquid chromatography/tandem mass spectrometry

## Abstract

A bovine milk reference material was produced containing amoxicillin with a target concentration at the maximum residue limit. For the evaluation and characterization of the reference material, an isotope dilution–liquid chromatography/tandem mass spectrometry method was developed and validated. This material was prepared by collecting milk samples from cows treated with an appropriate amoxicillin injection, followed by freeze-drying, dilution, and homogenization of the authentic samples. The amoxicillin reference material, prepared in accordance with ISO Guide 35, was homogeneous and stable. It was assigned a certified value of (4.10 ± 0.13) μg/kg with a relative standard deviation between 2.93 and 5.29%, which meets the maximum residue limit (MRL) requirement of 4.0 μg/kg established by the United States, the European Union, and China. This reference material can be used for method validation and provides internal quality control assurance for the detection of amoxicillin.

## Simple Summary

Antibiotics such as amoxicillin are commonly used to treat bacterial infections in dairy cows, but improper use can leave drug traces in milk. These residues can harm consumer health and disrupt dairy production. To ensure milk safety, regulatory authorities set strict limits on how much of these drugs can remain in milk. Laboratories need reliable reference samples—called reference materials—to accurately check their testing methods for these residues. In this study, we developed a stable and uniform freeze-dried milk powder sample containing a known amount of amoxicillin, matching the maximum allowed limit of 4 μg/kg. We used a highly accurate measurement technique involving mass spectrometry to confirm the sample’s content. The final reference material was shown to be consistent across different batches and stable over time. This product provides laboratories with a trustworthy tool for validating their detection methods and improving the accuracy of milk safety monitoring, thereby helping to protect public health and support the dairy industry.

## 1. Introduction

Nowadays, antibiotics are widely used to treat infections by bacteria in human beings and animals. The frequent use of antibiotics results in the appearance of residues in the form of their parent compound or metabolites of animal origin like milk, egg, meat and so on [1,2]. Veterinary drug residues in food products of animal origin cause increasing problems and become important content in food safety [3,4,5]. Amoxicillin (AMO), one of the most commonly used beta-lactam antibiotics, is employed as an efficient therapeutic agent to confront mastitis in lactating cows [6,7]. As a broad spectrum antibiotic in vitro, it acts by inhibiting the cross-linkage between the linear peptidoglycan polymer chains that make up the bacterial cell walls [8]. However, the improper use of AMO may lead to trace residue in milk, and the AMO residue not only causes problems in the dairy industry but also in public health, as it shows potential carcinogenic, teratogenic, and mutagenic effects at a high-dose level in clinical use [9,10]. AMO also may cause diarrhea, indigestion, nausea, vomiting, stomach pain, headache, fever, and sore throat. The maximum residue limit (MRL) of AMO in milk is 4 μg/kg by the EU Commission Regulation No 37/2010 [11] and Codex Alimentarius [12]. Many confirmatory methods for the determination of AMO in milk have been reported by means of liquid chromatography–tandem mass spectrometry (LC-MS/MS). For instance, Hajrulai-Musliu et al. employed LC-MS/MS to detect five antibiotics, including AMO, in milk, reporting recoveries of 70.83–109.00% with repeatability and reproducibility of up to 21.12% and 23.44%, respectively [13]. More importantly, instead of using fortified samples, a reference material (RM) and/or certified reference material (CRM) for AMO is needed for method validation and quality control. According to the EU Commission Implementing Regulation, CRMs are recommended for verifying the trueness of analytical methods [14]. In the literature, few reports can be referenced for the development of RM of β-lactams in milk. Only Spisso et al. reported an RM of benzylpenicillin in milk with an assigned value and uncertainty of (8.28 ± 0.52) μg/L. Other characteristics like homogeneity and stability testing were carried out and proved this reference material is capable for internal quality control or validation purposes [15].

This work details the development and characterization of an in-house reference material for AMO in lyophilized bovine milk. The preparation included the production of incurred material, freeze-drying, dilution, and mixing. The material was subsequently certificated for its homogeneity, stability, assigned value, and uncertainty.

## 2. Materials and Methods

### 2.1. Standards and Chemicals

HPLC-grade acetonitrile, methanol, and formic acid were obtained from Fisher Scientific Inc. (Pittsburgh, PA, USA). Ethanol, sodium hydroxide, and sodium dihydrogen phosphate were acquired from Sinopharm Group (Shanghai, China). Ultra-pure water was produced by the Milli-Q Plus water purification system from Millipore (Bedford, MA, USA). Oasis HLB solid phase extraction (SPE) cartridges (6 mL, 500 mg) were obtained from Waters (Milford, MA, USA). The syringe filter (0.2 μm) was supplied by Pall (Ann Arbor, MI, USA). The AMO standard was purchased from Sigma-Aldrich (St. Louis, MO, USA). Stock standard solution of AMO was prepared at 1 mg/mL by dissolving the compounds in 50% acetonitrile with water and stored at −20 °C. Internal standard AMO-d4 was obtained from Toronto Research Chemicals (Toronto, ON, Canada). The stock standard solution of AMO-d4 was prepared at 100 μg/mL by dissolving the compounds in water and stored at −20 °C. AMO sodium salt for animal injection was purchased from Yuanzheng Pharmaceutical Co., Ltd. (Shijiazhuang, China).

### 2.2. Determination of Amo in Lyophilized Bovine Milk

#### 2.2.1. Extraction and Purification

An aliquot of homogenized milk (2 g) was transferred to a 10 mL capped polypropylene centrifuge tube, and internal standard AMO-d4 was added to the milk to a final concentration of 10 ng/mL. After being vortexed for 3 min, the samples were kept at room temperature for 15 min. Then, 6 mL of ethanol was added for extraction by vortexing for 5 min, followed by centrifugation at 1150× *g* for 15 min at 4 °C. The supernatant was collected and evaporated by a rotary evaporator from Hangzhou Ruicheng Instrument Co., Ltd. (Hangzhou, China) to less than 1.5 mL. In total, 4 mL of sodium dihydrogen phosphate buffer (0.1 mol/L, pH 8.0) was added and vortex-mixed.

The mixture was subjected to an Oasis HLB cartridge, which was preconditioned sequentially with 3 mL of methanol, 3 mL of water, and 3 mL of sodium dihydrogen phosphate buffer (0.1 mol/L, pH 8.0). The SPE cartridge was then rinsed with 3 mL of water. The target compound was eluted with 4 mL of 90% acetonitrile-water followed by evaporation under nitrogen to approximately 0.4 mL. The extract was reconstituted to 1 mL with 0.1% formic acid, vortex-mixed for 1 min, and centrifuged at 18,407× *g* for 20 min at 4 °C. The supernatant was filtered through a syringe filter prior to LC-MS/MS analysis.

#### 2.2.2. Instrument Conditions

Chromatographic analyses were performed on a Waters Acquity ultra-performance liquid chromatography system and separations were achieved on a Waters CORTECS UPLC C_18_ column (2.1 mm × 50 mm i.d., 1.6 μm particle size) (Milford, MA, USA) with a column oven temperature of 35 °C. The injection volume was 10 μL. The mobile phases comprised 0.1% formic acid in water (solvent A) and 0.1% formic acid in acetonitrile (solvent B). The flow rate was 0.3 mL/min, with the following gradient program: 0–0.5 min, 99% A; 0.5–1.5 min, 99–60% A; 1.5–2.0 min, 60% A; 2.0–2.1 min, 60–2% A; 2.1–2.5 min, 2% A; 2.5–2.6 min, 2–99% A.; 2.6–4.5 min, 99% A.

The LC system was coupled to a triple quadrupole mass spectrometry (Waters) with an electrospray ionization (ESI) source in positive mode with the following parameters: capillary voltage, 3.0 kV; source temperature, 100 °C; desolvation temperature, 350 °C; desolvation gas flow, 600 L/h. Multiple reaction monitoring (MRM) was performed and the corresponding transitions, cone voltage, and collision energy are summarized in Table S1.

#### 2.2.3. Preparation of Incurred Material

Three healthy dairy cattle with a weight of almost 700 kg and daily milk production of about 40 kg were fed separately for one week. The cattle should be validated to have no mastitis and the milk should have no AMO residues. The blank sample of milk was collected during the isolation period. The injection of AMO was performed with 71 mg/kg bw for three days consecutively. The incurred milk samples were collected individually from each cow 15 min after injection and subsequently pooled into a single composite sample before storage at −80 °C.

#### 2.2.4. Preparation of In-House Reference Material

The frozen milk sample was lyophilized with a 72 h freeze-drying program and the ratio of freeze-dried matter to the frozen sample was determined at 16.5%, which was used for the reconstitution of the final material. The freeze-dried-incurred and blank milk samples were converted to powder by grinding and sieving, respectively. The initial concentration of AMO was determined using the proposed LC-MS/MS method. Appropriate amounts of the incurred sample were diluted with blank samples and homogenized in a Turbula mixer in order to obtain the target concentration at MRL of 4 μg/kg. After dilution, the material was bottled in 5 g portions and the samples were stored at −80 °C for the evaluation of homogeneity and stability. A total of 500 bottles were prepared for the RM.

#### 2.2.5. Homogeneity and Stability

For the homogeneity test of RM, 15 bottles of the product were selected randomly and triplicate measurements were performed by means of the proposed LC-MS/MS method. Analysis of variance was carried out to estimate between-bottle variation. The short-term and long-term stability studies were set up and performed using the isochronous approach [16]. For the short-term stability study, 60 samples were selected randomly and 5 samples each were stored at +20 °C, +4 °C, and −20 °C conditions for 1, 2, 3, and 4 months, respectively. The evaluation of long-term stability was scheduled for 0.5, 1, and 2 years at −20 °C and −80 °C, and the results of the test will not be reported in this work.

#### 2.2.6. Characterization of the Reference Material

A collaborative study was organized, involving five participants on the basis of using the proposed LC-MS/MS method. The five laboratories were all accredited according to ISO 17025 [17] and experienced in the analysis of AMO in milk. Each laboratory received four bottles of candidate RM and was required to submit eight analytical results of reconstituted material. Reconstitution of the lyophilized milk powder was prescribed by using 0.33 g powder to be mixed with 1.67 g of water. Laboratories exclusively applied matrix-matched calibration and the isotope-labelled internal standard. The results were checked for outliers using the Grubbs test [18].

## 3. Results and Discussion

### 3.1. Method Optimization

In the previous reports, 100% acetonitrile was utilized for extracting AMO in milk samples [19,20,21]. Meanwhile, 75% acetonitrile was used as the extractant of AMO in milk, according to the standard method of China and other literature [22]. Ethanol was also applied for the extraction of AMO in milk samples [23,24]. We evaluated the three different extractants with respect to their efficiency in extracting AMO from milk samples. As a result, 100% acetonitrile and 75% acetonitrile demonstrated no more than 50% recovery, which was relatively lower than the extraction efficiency of ethanol. During the purification process, the eluting solution was optimized using 10% to 100% acetonitrile separately to elute the analyte from the cartridge. According to the elution curve of AMO, 90% acetonitrile was adopted for the best recovery.

### 3.2. Method Validation

**Figure 1:**
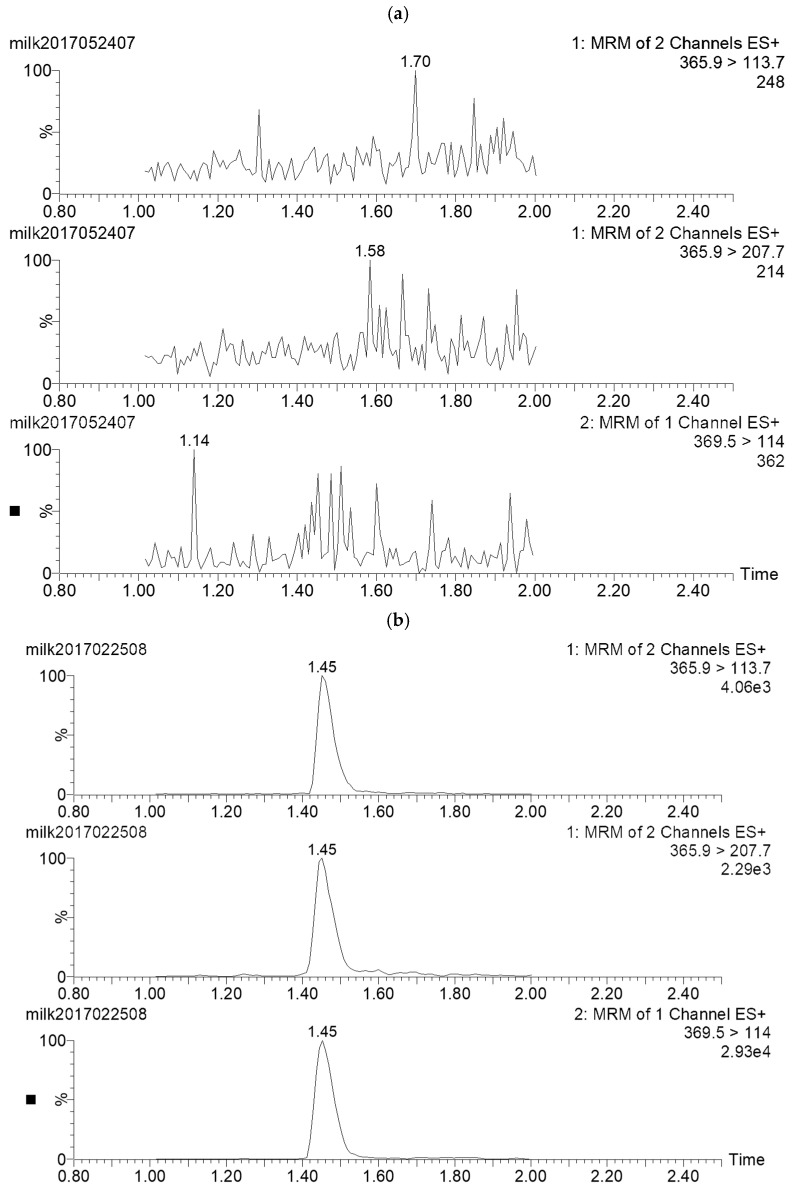
UHPLC-MS/MS chromatograms of the fortified milk sample at the LOQ level. (**a**) blank sample; (**b**) fortified sample.

The selectivity, linearity, accuracy, precision, limit of detection (LOD), and limit of quantification (LOQ) were assessed during the validation of the proposed method. No interfering peaks were observed at the retention times of the AMO and internal standard in the blank samples. Good linearity was obtained, with coefficients of determination (r^2^) higher than 0.999 for AMO in the matrix calibration within the range of 2–500 μg/kg. For the evaluation of method accuracy and precision, fortified samples were prepared in six replicates at four levels (2, 4, 8, 50 μg/kg) on three different days. As shown in Table S2, the mean recoveries were between 90.9% and 100.6%, with intra-day and inter-day relative standard deviation (RSD) lower than 8.0% and 7.0%, respectively, indicating the good accuracy and repeatability of the method. Typical MRM chromatograms of blank and fortified bovine milk samples at the LOQ level are shown in Figure 1. LOD and LOQ, defined as the concentration with a signal-to-noise ratio of 3 and 10, were 0.5 μg/kg and 2 μg/kg for AMO in milk, respectively, which were sensitive enough for the determination of AMO at a target concentration of 4 μg/kg. The recovery and precision of the proposed method were comparable to those of a previously reported method that also employed internal standard quantification, whereas the sensitivity was superior [25].

### 3.3. Preparation of In-House Reference Material

During the freeze-drying process, the determination of pre-freezing temperature is crucial, which is related to the eutectic temperature of the milk sample. In this study, we used a multimeter and thermometer to determine the eutectic temperature of the milk sample. As presented in Figure S1, the eutectic temperature of milk was approximately −20 °C. For rapid and complete freezing of the sample, −30 °C (10 °C lower than the eutectic temperature) was selected as the temperature for pre-freezing. Another critical factor of the lyophilization is the thickness of the sample, which will affect the moisture content of milk powder. Usually, the moisture content of commercial milk powder should be controlled below 5% for long-term storage. We compared the moisture content of lyophilized samples resulting from different thicknesses (10 mm, 15 mm, and 20 mm). As expected, the thickness of the sample was closely related to the moisture content, and the thickness of 15 mm was adopted in this work (Figure S2).

### 3.4. Homogeneity and Stability

A homogeneity test was performed with the proposed LC-MS/MS method for the selected 15 bottles, and the results are summarized in Table 1.

**Figure 2:**
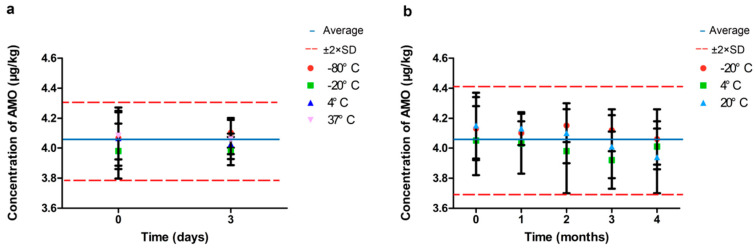
Short-term (**a**) and long-term stability (**b**) graphs of AMO CRF. Error bars represent ±SD (*n* = 5).

We followed the scheme described in Section 7.8 and Annex B.3 of the ISO Guide 35 [26]. The average AMO concentration of the selected bottles was 4.11 μg/kg with a between-bottle standard deviation of 0.13 μg/kg. The RSD among bottles was 3.16%, indicating good between-bottle homogeneity of the RM. The short-term stability was carried out and no degradation was observed for the tested conditions (+20 °C, +4 °C, and −20 °C) for up to six months (Figure 2).

We further evaluated the measurements of stability test according to the Annex B.5 of the ISO Guide 35 [26], and the results are summarized in Table 2.

The stability of the RM within four months was confirmed since all the slopes were insignificant.

### 3.5. Characterization and Uncertainty

As summarized in Table 3, the mean certificated values of the five laboratories were 4.01–4.16 μg/kg with and RSD between 2.93 and 5.29%. Data were subjected to statistical analysis using the Grubbs test and a few outliers were detected. The detected outliers were scrutinized and the values were retained because no apparent technical reason was obtained for their exclusion. Therefore, the property value of the developed RM was 4.10 μg/kg. Calculation of the overall expanded uncertainty (*u_CRM_*) should consider uncertainty contributions from characterization (*u*_char_), homogeneity (*u*_bb_), and long-term and short-term stability (*u*_lts_, *u*_sts_) [26]. Since the long-term stability test had not been completed, the uncertainty associated with the property value of the RM was expressed as

$$u_{RM}=\sqrt{u_{bb}^{2}+u_{sts}^{2}+u_{char}^{2}}$$

and the calculated value was 0.13 μg/kg. The RM developed in this study exhibits a lower uncertainty than the previously reported benzylpenicillin RM, demonstrating its high reliability [15].

## 4. Conclusions

A new in-house reference material has been produced for amoxicillin in a lyophilized bovine milk matrix. Its characterization was performed using a specifically developed and reliable LC-MS/MS method, yielding an assigned value of (4.10 ± 0.13) μg/kg. The reference material demonstrated satisfactory homogeneity and stability, confirming its suitability for the method validation and internal quality control of amoxicillin detection.

## Figures and Tables

**Table 1:** Homogeneity result of the reference material.

Bottle No.	Result No. 1 (μg/kg)	Result No. 2 (μg/kg)	Result No. 3 (μg/kg)	Average (μg/kg)
1	4.12	4.03	4.16	4.10
2	4.20	4.11	4.23	4.18
3	4.21	4.03	4.17	4.14
4	4.33	4.21	4.23	4.26
5	4.06	4.01	4.09	4.05
6	4.17	4.10	4.21	4.16
7	3.99	3.89	3.86	3.91
8	3.90	3.85	4.01	3.92
9	4.22	4.38	4.33	4.31
10	4.20	4.11	4.23	4.18
11	3.90	3.93	3.90	3.91
12	4.38	4.24	4.40	4.34
13	4.00	3.99	4.05	4.01
14	4.12	4.18	4.15	4.15
15	3.93	4.09	4.11	4.04
Between-bottle standard deviation, *s*_bb_	0.13 μg/kg (3.16%)			

**Table 2:** Short-term stability of the reference material.

Storage Temperature	*b*_1_	*b*_0_	*s* (*b*_1_)	*t*_0.95,3_·*s* (*b*_1_)
−20 °C	0.00042	4.1092	0.013907	0.04359
+4 °C	−0.004683	4.0902	0.01822	0.05794
+20 °C	0.006177	3.9970	0.02102	0.06684

*b*_1_: slope; *b*_0_: intercept; *s* (*b*_1_): uncertainty associated with the slope; *t*_0.95,3_*·s* (*b*_1_): *n* − 2 degrees of freedom and 95% level of confidence.

**Table 3:** Summary of characterization.

Laboratory	Experimental Results (μg/kg)								Mean (μg/kg)	RSD (%)
Lab 1	4.14	4.02	4.20	4.01	3.89	3.88	4.31	4.17	4.08	3.75
Lab 2	3.90	3.77	4.12	4.32	3.95	4.01	4.15	3.86	4.01	4.46
Lab 3	4.05	4.28	4.08	3.96	4.30	4.21	4.13	4.25	4.16	2.93
Lab 4	3.85	4.38	4.02	4.27	4.13	4.26	3.79	4.30	4.13	5.29
Lab 5	3.86	4.41	3.95	4.21	4.17	4.22	3.80	4.26	4.11	5.22

## Data Availability

The original contributions presented in this study are included in the article/Supplementary Material. Further inquiries can be directed to the corresponding authors.

## Supplementary Materials

The following supporting information can be downloaded at: https://www.mdpi.com/article/10.3390/vetsci13020193/s1, Figure S1: The eutectic point of the sample; Figure S2: Effect of sample thickness on freeze-drying; Table S1: Transitions and optimized conditions for MS/MS analysis; Table S2: Recovery and precision.
