# Supplementary material for: Preparation of a Lyophilized Bovine Milk Reference Material for Quality Control of Amoxicillin Detection
Source: Vet Sci. 2026 Feb 15;13(2):193. doi: 10.3390/vetsci13020193 (PMC12945065; doi:10.3390/vetsci13020193)
Supplement: Supplementary file 1 [file vetsci-13-00193-s001.zip › vetsci-4060166-supplementary.pdf]

# Supplementary Material

# Preparation of a Lyophilized Bovine Milk Reference Material for Quality Control of Amoxicillin Detection

**Shulin Wei [1,†], Wei Zhang [1,†], Decheng Suo [1], Peilong Wang [1], Kang Ma [2,*] and Ruiguo Wang [1,*]**

[1] Institute of Quality Standards and Testing Technology for Agro-Products, Chinese Academy of Agricultural Sciences, Beijing 10081, China; weishulin@caas.cn (S.W.); zhangwei@caas.cn (W.Z.)
[2] National Institute of Metrology of China, Beijing 10088, China
* Correspondence: makang@nim.ac.cn (K.M.); wangruiguo@caas.cn (R.W.); Tel.: +86-10-82106576 (R.W.)
† These authors contributed equally to this work.

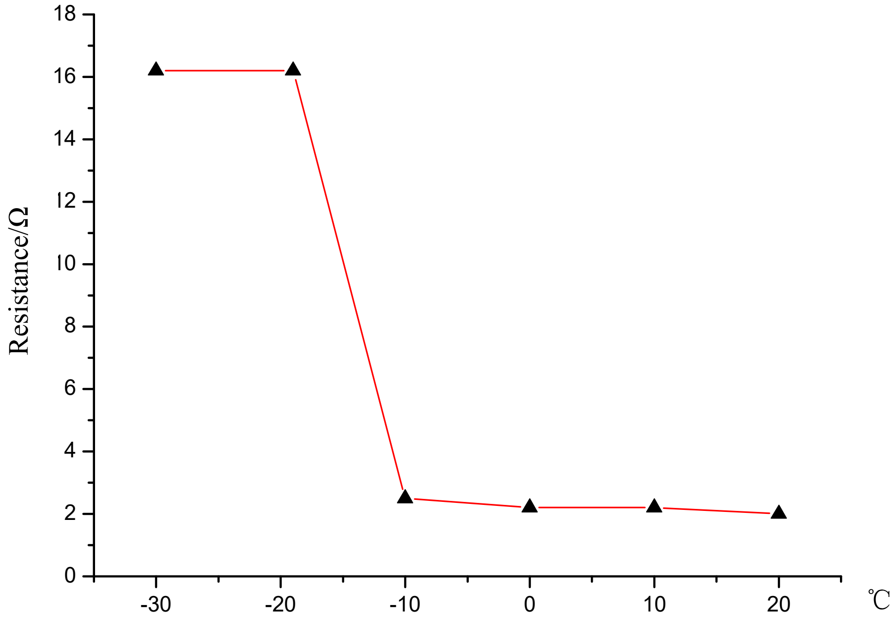


**Figure S1** The eutectic point of the sample

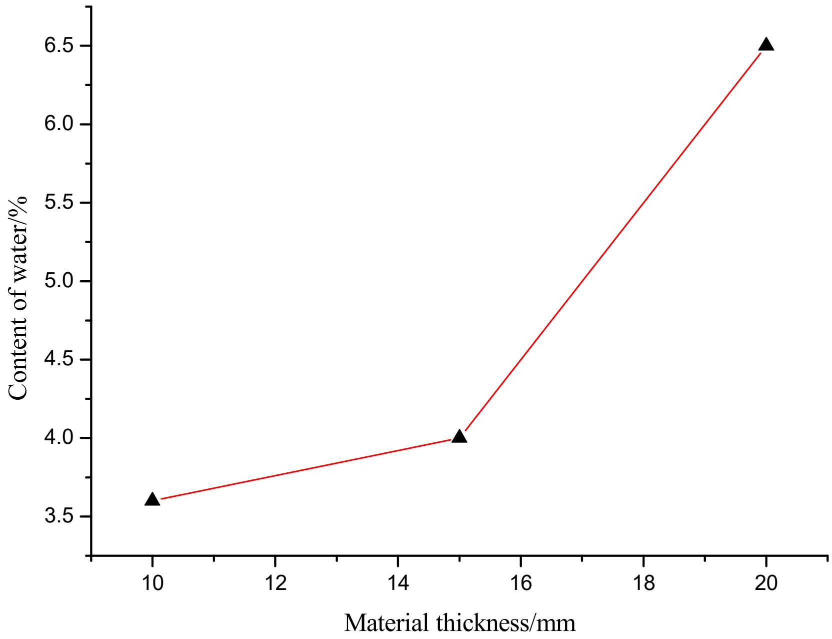


**Figure S2** Effect of sample thickness on freeze-drying

**Table S1** Transitions and optimized conditions for MS/MS analysis

| Analytes | Retention time (min) | Precursor ion (*m/z*) | Product ions (*m/z*) | Cone voltage (V) | Collision energy (eV) |
|---|---|---|---|---|---|
| AMO | 1.44 | 366.5 | 113.7 [a] | 14.00 | 20 |
| | | | 207.7 | 14.00 | 14 |
| AMO-$d_4$ | 1.44 | 369.5 | 114.0 | 15.00 | 18 |

[a] Ions for quantification

**Table S2** Recovery and precision

| Spiked level (μg/kg) | Recovery (%) | | | | | | Intra-day mean recovery (*n*=6, %) | Inter-day mean recovery (*n*=18, %) | Intra-assay RSD (*n*=6, %) | Inter-assay RSD (*n*=18, %) |
|---|---|---|---|---|---|---|---|---|---|---|
| 2 | 94.89 | 93.96 | 102.11 | 85.00 | 84.00 | 95.10 | 92.51 | 92.85 | 7.4 | 6.2 |
| | 95.44 | 87.18 | 95.89 | 88.03 | 85.00 | 94.00 | 90.92 | | 5.2 | |
| | 94.00 | 87.63 | 90.18 | 102.48 | 98.02 | 98.38 | 95.11 | | 5.8 | |
| 4 | 97.30 | 98.50 | 90.33 | 100.50 | 88.95 | 91.23 | 94.46 | 95.93 | 5.1 | 4.4 |
| | 97.60 | 97.00 | 99.21 | 101.5 | 91.50 | 92.30 | 96.51 | | 4.1 | |
| | 89.56 | 95.60 | 99.65 | 97.22 | 96.30 | 102.52 | 96.80 | | 4.5 | |
| 8 | 102.20 | 99.17 | 101.20 | 104.10 | 94.92 | 102.19 | 100.59 | 97.84 | 3.2 | 7.0 |
| | 105.79 | 93.70 | 104.24 | 91.64 | 104.70 | 103.04 | 100.51 | | 6.1 | |
| | 84.97 | 91.00 | 99.36 | 86.50 | 88.42 | 104.27 | 92.94 | | 8.0 | |
| 50 | 95.06 | 101.50 | 102.50 | 98.04 | 93.96 | 105.10 | 99.36 | 96.92 | 4.4 | 5.3 |
| | 96.96 | 92.51 | 96.37 | 90.00 | 86.59 | 88.00 | 91.73 | | 4.7 | |
| | 98.10 | 101.35 | 100.38 | 99.38 | 101.90 | 96.90 | 99.66 | | 1.9 | |
